# Method for Indoor Seismic Intensity Assessment Based on Image Processing Techniques

**DOI:** 10.3390/jimaging11050129

**Published:** 2025-04-22

**Authors:** Jingsong Yang, Guoxing Lu, Yanxiong Wu, Fumin Peng

**Affiliations:** Department of Information Science and Control Engineering, Institute of Disaster Prevention, Sanhe 065201, China; yangjingsong@cidp.edu.cn (J.Y.); weluckin@163.com (G.L.); pengfumin2024@163.com (F.P.)

**Keywords:** seismic intensity assessment, image segmentation, artifact reaction, camera shake compensation, the depth of field area is established

## Abstract

The seismic intensity experienced indoors directly reflects the degree of damage to the internal structure of the building. The current classification of indoor strength relies on manual surveys and qualitative descriptions of macro phenomena, which are subjective, unable to capture real-time dynamic changes in the room, and lack quantitative indicators. In this paper, we present the Image Evaluation of Seismic Intensity (IESI) method, which is based on image processing technology. This method mainly evaluates the degree of responses from objects by identifying the percentage of movement of different types of objects in images taken before and after an earthquake. In order to further improve the recognition accuracy, we combined the camera vibration degree and the object displacement between images to correct the generated earthquake intensity level estimation, so as to achieve the rapid assessment of an earthquake’s intensity indoors. We took, as an example, 29 sets of seismic data from different scenarios. We used the IESI method to evaluate the seismic intensity of these scenarios. Compared with the seismic intensity evaluation results obtained by the Post-disaster Sensor-based Condition Assessment of Buildings (PSAB) and the Image-based Seismic Damage Assessment System (IDEAS) methods, the accuracy of the IESI method was higher by more than 30%, and its accuracy reached 97%. The universality of the IESI method in different indoor scenarios was demonstrated. In a low-intensity evaluation experiment, the accuracy of the IESI method also reached 91%, which verifies the reliability of the IESI method in low-intensity regions.

## 1. Introduction

Through the extensive deployment of monitoring equipment in different scenarios and areas, we can obtain first-hand earthquake video data. Combined with computer vision technology, information about the disaster is quickly obtained, the image processing algorithm is used to analyze the reaction of objects, and the earthquake’s intensity indoors is evaluated according to the information on the movement of objects. The indoor object response refers to the varying degrees of movement exhibited by objects within a room as a result of an earthquake, reflecting the level of damage within that particular space. It serves as a crucial scientific basis for assessing the disaster situation and formulating rescue plans, as well as the primary means of evaluating the seismic intensity experienced indoors [1,2,3]. In the latest version of the “China Earthquake Intensity Scale”, released in 2020 [4], a detailed definition is provided for the correspondence between the degree of the object response within a scene and the seismic intensity level.

After an earthquake, the situation inside buildings becomes complex, making it challenging to directly capture the extent of the damage. Currently, the seismic intensity experienced indoors is mainly assessed through manual evaluation based on postearthquake indoor image data and residents’ verbal descriptions [5,6]. This method offers high accuracy and the ability to assess various scenarios, but it has limitations in terms of timeliness and cost-effectiveness. Erign. T et al. [7] proposed a sensor-based framework for post-disaster building condition assessment (PSAB), which involves deploying a large number of sensors to capture the movement of objects within the building for assessing the extent of damage. While this approach allows for the more accurate capture of information regarding the movement of indoor objects, the deployment of sensors is costly and difficult to implement on a wide scale, and relying solely on the degree of objects’ movement results in a lower accuracy in assessing the seismic intensity. Ning Chaolie et al. [8] established a probabilistic seismic loss assessment model for non-structural components, enabling the analysis of the damage rates of these components under different earthquake magnitudes. However, these damage rates cannot be directly correlated with seismic intensity. In recent years, with the continuous improvement of monitoring systems, a large number of indoor seismic videos have been captured [9,10], providing convenient conditions for studying image-based seismic intensity assessment methods. Currently, the assessment of object response levels indoors heavily relies on prolonged observations by professionals through various monitoring videos, which can lead to fatigue and a decrease in accuracy [11]. Edward-TH-CHU et al. [12] proposed the Image-based Seismic Damage Assessment System (IDEAS), which quantifies the degree of object response and evaluates seismic intensity based on the degree of objects’ movement, using the area variance among objects to identify the seismic intensity. This method can accurately assess scenes with lower levels of damage. However, it faces challenges in accurately identifying the seismic intensity experienced in rooms with significantly different object area variances in cases of more severe damage, and it is susceptible to abnormal images, leading to a decreased evaluation accuracy. In summary, there is currently a lack of methods for processing abnormal images and assessing the entire range of object response intensities in order to rapidly evaluate the seismic intensity experienced indoors.

This study focuses on the analysis of object responses and seismic intensity assessments in individual rooms within a short period of time after an earthquake. The specific workflow is as follows: First, feature point detection algorithms are utilized to extract stable regional feature points from pre- and postearthquake images. Based on the feature point information, abnormal images are eliminated, and camera shake is compensated for [13]. The SAM and YOLOv8 models are combined to detect and remove human bodies from the pre- and postearthquake images, followed by image restoration techniques [14,15,16]. Simple depth estimation is performed on the images to classify objects based on their sizes. Then, different segmentation and matching algorithms are used depending on the complexity of the images [17,18,19,20]. Objects’ movements between the pre- and postearthquake images are calculated, and the degree of object response is quantified by considering the quantity, size, and displacement of objects. The seismic intensity is evaluated accordingly. Finally, the level of camera shake and the number object falls in the images are analyzed to adjust the seismic intensity assessment. To validate the accuracy of this method, a total of 29 sets of real earthquake video data from Shandong Dezhou (5.5 magnitude), Sichuan Luding (6.8 magnitude), and Syria (7.8 magnitude) were selected, covering common scenes such as residential areas, offices, shopping malls, and factories. The evaluation results are compared with other existing methods, demonstrating a significantly higher accuracy. Furthermore, the seismic intensity assessment results for the Dezhou earthquake are compared with the intensity map published by the China Earthquake Administration, showing consistent intensity evaluation ranges. The thesis mainly elaborates from the following aspects:

Section 1: Introduction. This section summarizes the urgency and necessity of indoor seismic intensity evaluation, analyzes the limitations of existing methods of rapid evaluation, and puts forward an image-based evaluation method to improve the efficiency and accuracy. The purpose and significance of the study are clarified.

Section 2: Data Description and Preprocessing. This section focuses on pre/postearthquake image acquisition and enhancement techniques. It includes anomaly detection, depth compensation for specific scenes, and object categorization based on size to improve the algorithmic accuracy.

Section 3 and Section 4: Area-Based Object Classification and Acquisition of Object Movement and Seismic Intensity Assessment. This section introduces the integrated image processing algorithm (segmentation and matching), which analyzes the degree of the object response for indoor seismic intensity assessment. The evaluation results are revised.

Section 5: Simulation Experiments and Analysis. This section validates the proposed IESI framework using indoor seismic video datasets across four scenarios. It demonstrates the workflow’s reliability through data processing and algorithm verification.

**Figure 1:**
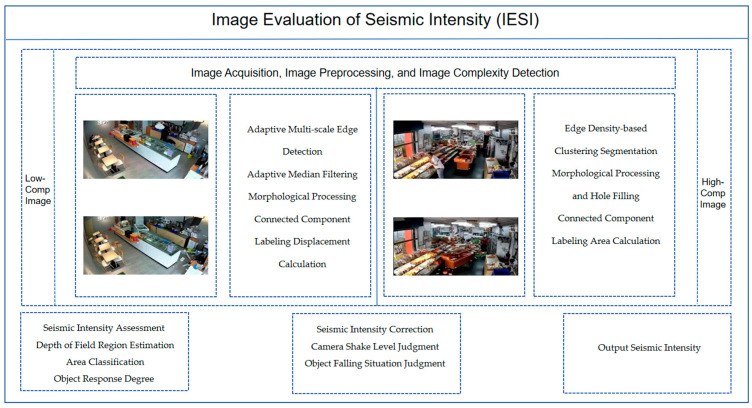
Technology roadmap.

Section 6: Conclusions. This section summarizes the IESI framework and highlights innovations in the image-based intensity assessment, as well as potential improvements. The overall technical roadmap is shown in Figure 1.

## 2. Data Description and Processing

### 2.1. Image Acquisition and Preprocessing

After an earthquake, due to the effects of various factors, there were various abnormal conditions in the images taken. In order to obtain the images of the rooms taken before and after the earthquake with the most complete information, it was necessary to select a suitable image preprocessing algorithm and workflow. The acquisition and enhancement of images of the rooms taken before and after the earthquake were carried out, and some abnormal conditions were preprocessed. The preprocessed images were processed with regard to their depth of field, and the objects were classified.

#### 2.1.1. Video Acquisition and Pre/Postearthquake Image Selection

To ensure data reliability, real earthquake videos were downloaded from platforms (e.g., Baidu (v13.16.0.10), Bilibili (v6.59.0), and YouTube (v17.18.34)). The videos were standardized to 60 fps. The extract-frames function in Python(Python3.10) was utilized to achieve video framing. In order to ensure the integrity of the information regarding the movement of objects before and after the earthquake, the last frame taken before the earthquake began was treated as the image before the earthquake, and the first frame taken after the earthquake was completely over (no more object movement occurred) was treated as the image after the earthquake. Raw images often contained watermarks and black borders, so these were removed using Photoshop (Photoshop2021). Additional steps included enhancing the sharpness of the images to improve feature visibility and standardizing the resolution. The images were resized to 800 × 600 pixels for algorithm compatibility. The images were saved in JPG format for subsequent processing.

#### 2.1.2. Brightness Compensation

Brightness compensation is crucial for earthquake images captured by surveillance systems, which often suffer from uneven illumination being caused by factors like abnormal lighting and dust, leading to overexposed or underexposed areas that obscure object features. The core approach involved balancing the brightness distribution between overly bright and dark regions. Compared to other methods, adaptive histogram equalization (AHE) offers advantages, such as a low computational complexity, the preservation of local details, and natural contrast enhancement, without requiring prior information. AHE achieves this by expanding the grayscale coverage based on pixel distribution characteristics from the image’s histogram. The formula for adaptive histogram equalization is shown in Equation (1).

H(i,j)=∑[0,i]∑[0,j]I(i,j)

C(i,j)=(H(i,j)−H_min)/(i×j−1)

(1) I′(x,y)=C(I(x,y))×(L−1)

**Figure 2:**
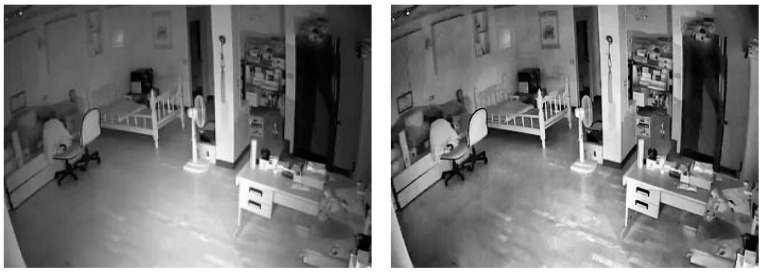
Images before and after adaptive histogram equalization.

To achieve adaptive histogram equalization, it is necessary to preset the size of a local region, divide the image into different regions according to size, and perform block histogram equalization for all regions, and then perform an overall histogram equalization for the whole image after regional histogram equalization; the preset local area size was 200 × 150, and the image was divided into 16 blocks. In Equation (1), i × j represents the size of the local area, I(i, j) represents the pixel gray value, H(i, j) represents the pixel distribution in the local area, H_min represents the lowest gray level in the local area, C(i, j) represents the cumulative distribution function, and (L − 1) represents the gray level. I′(x, y) is the pixel after equalization. The image, before and after adaptive histogram equalization was performed, is shown in Figure 2.

From the before-and-after comparison in Figure 2, it is evident that the luminance anomalies in the left, upper, and upper-right corners of the room, which are susceptible to light shadows, have been greatly improved. The outline of the objects, the edges, and the texture are clearer.

#### 2.1.3. Bilateral Filtering

To obtain the information regarding the movements of objects, the image needed to be divided into the background and the foreground, and the structural components, such as ceilings and walls, which do not change in the low-intensity area, are regarded as the background, and the remaining non-structural components that may move are regarded as the foreground. The grayscale similarity of the background area in the image is very high. In order to reduce the noise, blur the background, and highlight the foreground, a two-sided filter was used to reduce the image noise.

**Figure 3:**
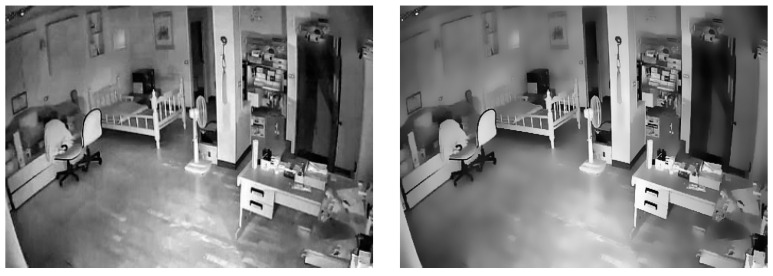
Before and after bilateral filtering.

Compared with other filters, a double-sided filter can effectively retain the edge information and detail information in the image when smoothing the image, making the information in the filtered image clearly visible [21]. The working principle of the two-sided filter is to obtain a weighted average of the neighborhood pixels of the center pixel according to the spatial distance and gray similarity of the pixel. The closer the distance and the higher the gray pixel, the higher the weight of the pixel, so that the edge information can be retained and the background can be blurred. The bilateral filter also has the advantage of high stability. The bilateral filter uses a window size of 5 × 5 and is implemented based on the bilateral filter function in Python Opencv 4.7.0. The image, before and after bilateral filtering was performed, is shown in Figure 3.

From Figure 3, it can be found that the background area is partially blurred, the foreground area is prominent, and the texture mutation is more obvious.

#### 2.1.4. Local Significance Detection

In the image after bilateral filtering was performed, it can be seen that filtering has removed most of the edge noise and simply highlighted the foreground area. Then, a local significance detection algorithm was used to process the image after bilateral filtering to further blur the background and highlight the foreground [22].

**Figure 4:**
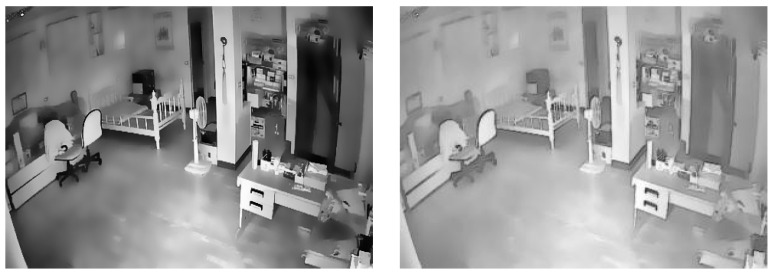
Images before and after local significance detection.

Compared with other foreground-highlighting algorithms, local saliency detection is calculated based on local contrast and gray level information and does not require a global analysis of the entire image. While highlighting the foreground, it can also increase the contrast and improve the accuracy of the subsequent foreground segmentation. Local significance detection works by calculating the contrast of each pixel with the surrounding area via weighted processing. It calculates the contrast between the central region and the surrounding region, takes the average value between the central contrast and the surrounding contrast, and then calculates the difference between the two as the significance value of the pixel. Local significance detection also has the advantages of robustness and high universality. The weighting method was set as Gaussian weighting, and the threshold of the binary screening was 100. The image, before and after local significance detection was performed, is shown in Figure 4.

As can be seen from Figure 4, the background area is blurred, and the foreground area is partially prominent, but the phenomenon of the partial blurring of foreground objects can be seen. In the future, the segmentation threshold should be appropriately reduced in the segmentation algorithm to avoid missing the detection of objects.

### 2.2. Abnormal Image Preprocessing

After an earthquake occurs, abnormal images are produced due to poor image quality, camera shake, human movement, and other factors. In cases where heavy furniture is displaced, lighting is damaged, and structural components are affected, poor-quality images are often captured. This kind of image is not suitable for the analysis of the target: the degree of response. Camera shake causes pixel displacement throughout the image, which results in the false detection of the movement of all objects and even the background. This greatly affects the accuracy of seismic intensity assessments. There is often human movement in images taken before and after an earthquake, which is easy to misidentify as the target movement during the target detection, resulting in the incorrect analysis of the target movement before and after the earthquake. These anomalies need to be pre-processed before the motion of objects in the room can be analyzed.

#### Remove Low-Quality Images

Low-quality images are manifested when the vast majority of heavy furniture has moved, the lights are damaged, and the structural components are damaged. Such images are usually taken in areas of high intensity, where earthquakes’ intensity is greater than 9 degrees. As can be seen from the description of the seismic intensity table, the intensity range of an indoor object’s reaction can be evaluated as 5–9 degrees, so the seismic intensity of such images cannot be evaluated, and the low-quality images need to be removed first. Because too much object information is lost in low-quality images, there are usually very few stable feature point pairs detected, and there are a large number of mismatched point pairs. Therefore, the low-quality images can be eliminated according to the matching of stable feature points in the images before and after the earthquake.

Seismic images are susceptible to brightness changes, camera shaking, and other factors. In order to detect the feature points in the stable region, the AKAZE algorithm [23] with illumination, rotation, and scale invariance was selected to detect the feature points. A nonlinear scale space was constructed from low to high by the scale decomposition of the image, and the feature points on the scale pyramid of each layer were detected by a Hessian matrix. When all the points on the adjacent scales are corner points, this point is determined to be a stable feature point. Finally, the gradient diffusion method was used to obtain the gradient direction and the size of the local region, and a feature point descriptor was generated according to the gradient direction of the features in the region.

The K-nearest neighbor matching algorithm [24] was adopted to match feature points, and the matching feature points were judged according to the distance difference between the nearest neighbor and the second-nearest neighbor of the matching feature points. In this way, the accuracy of the matching feature points was improved. The K-nearest neighbor matching algorithm has a simple structure, a fast operation speed, and high stability. We set the difference threshold coefficient to 0.8.

To detect the feature points in the stable region, it was necessary to filter out the abnormal points in the middle distance of the matching feature points. The traditional feature point screening algorithm has the problem of a low screening rate. The homologous matrix model (RANSAC) algorithm [25] was used to randomly select feature points to build a model, compare the remaining feature points with the model, and calculate the difference between them. When the difference was less than the threshold, the points were classified as internal points, and the model was gradually updated. After reaching the set number of iterations, the model with the most internal points detected was the optimal model. Based on this model, the stable feature point pairs approximating the background region were screened out, and the distance between the matching feature points was calculated.

We filtered outliers based on the distance between feature points. Due to the large distance between the outlier matching points, traditional outlier filtering methods [26] based on quartiles may exclude smaller outliers. Therefore, the single threshold quartile method was used to calculate the high threshold, eliminating the outliers outside the specified range.

(2) iqr=q3−q1,G=q3+k×iqr

In Equation (2), q3 represents the upper quartile, q1 represents the lower quartile, k is the coefficient, and G is the high threshold. The matching point pairs with feature point distances of greater than the high threshold were filtered out. In this study, a total of 109 sets of real earthquake images obtained from various major video websites were used to statistically analyze the relationship between the camera shake level and the calculation of feature point distances. When the distance between feature points ranges from 0 to 2.229, there is no shaking; from 2.229 to 12.229, there is slight shaking; and from 12.229 to the full width, there is obvious shaking.

To eliminate low-quality images, it was necessary to perform stable feature point detection on the filtered data. The detection process was as follows: Firstly, the data is sorted and divided into the first 80% and the last 20% of the data. The mean value “a” of the first 80% of the data and the maximum value “b” of the last 20% of the data are calculated. Then, considering the threshold values obtained from the statistical analysis of the camera shake level in Table 1, the following criteria are set to determine the anomalous data: When a < 2.229, the camera has minimal or no shake. If b − a > 2a, the image is removed. When 2.229 ≤ a < 12.229, the camera experiences slight shaking. If b − a > a, the image is removed. When a ≥ 12.229, the camera exhibits significant shaking. If b − a > 0.5a, the image is removed.

**Figure 5:**
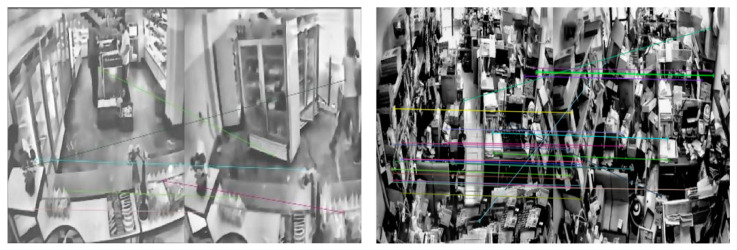
Comparison of matching images between low-quality images and filterable images.

As shown in Figure 5, after filtering out abnormal feature points, the distance between the feature points in the filterable images no longer have any abnormal values, whereas the filtering results of low-quality images still contain some abnormal points. At this point, the low-quality images were discarded, and the filterable images were transmitted to the camera shake compensation module.

### 2.3. Camera Shake Compensation

Camera shaking often occurs after an earthquake. Camera shaking does not cause changes along the z axis, but only causes rotation, translation, and other deformation between images taken before and after the earthquake. Image processing is essentially pixel matrix processing, so the camera shake compensation problem can be abstracted into an image registration problem based on affine transformation matrix [13]. First, the rotating, translational homogeneous equation was constructed according to the set of characteristic points taken before and after the earthquake, and the mean variance of the Euclidian distance between point pairs was used as the loss function. The parameters of the affine transformation matrix were adjusted by the least squares method to minimize the loss function value. The affine transformation matrix between the images was calculated to obtain the rotation matrix and the amount of displacement between the images. Then, we implemented camera shake compensation.

The principle of utilizing the affine transformation matrix to calculate rotational translation is as follows: According to the coordinate information of P = (x, y) and P’ = (x’, y’) from before and after the earthquake, the affine transformation equation is constructed, where P = (x, y) is the coordinate of the feature points before the earthquake, and P’ = (x’, y’) is the coordinate of the feature points after the earthquake. Rotation transformation refers to taking a rotation center as a reference point and rotating the point according to a certain corner point; translation transformation refers to moving the point according to a certain translation vector. The form of the affine transformation equation constructed by this method is as follows:

(3) $$\begin{array}{c} X=Ax+By+C\\ Y=Dx+Ey+F \end{array}$$

In Equation (3), [x, y] refers to the coordinates of feature points before and after the earthquake, [X, Y] refers to the coordinates of feature points after the earthquake, and [C, F] refers to the translation quantity of feature points in the direction of x and y, respectively, before and after the earthquake, and refers to the rotation matrix. After constructing the affine transformation matrix, the Euclidean distance square was set as the error function, and the original point was brought into the affine transformation matrix. The square distance between the calculated target point and the real target point was the object, and the average error of all stable feature points was calculated, and the error was minimized by adjusting the affine transformation matrix parameters (translation amount, rotation angle). The gradient descent method was used to iteratively update the parameter values, and the optimal solution was obtained step by step. The rotation matrix was substituted into Equation (3) to calculate the rotation components between images.

(4) $$\theta =-{tan}^{-1}(\frac{R_{1,0}}{R_{0,0}})\times \frac{180}{\pi }$$

In Equation (4), θ is the rotation angle, and *R*_1,0_ and *R*_0,0_ represent the elements in the first column of the second row and the first column of the first row of the rotation matrix, respectively. Finally, the calculated rotation translation quantities were combined with the coordinates of the postearthquake stability characteristic points to perform inverse rotation translation processing on the postearthquake image.

**Figure 6:**
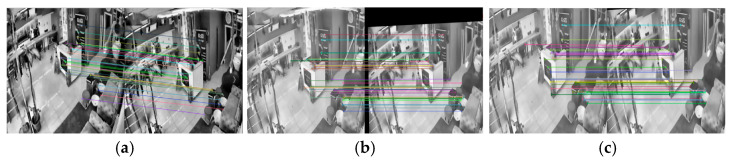
Camera shake compensation. (**a**) original image; (**b**) shake compensation; (**c**) black edge removal and image cropping.

As shown in Figure 6a,b, the stable regions of interest between the images before and after the earthquake were successfully matched. As can be seen from Figure 6b, after the inverse transformation of the postearthquake image, black edges were often generated at the left and upper edges of the image. It was found through observation that the black edges are located in background areas, such as walls, the ground, and the ceiling, which do not affect the subsequent acquisition of indoor object movement and can be directly removed. To achieve this, a threshold-based black edge removal algorithm was used: The number of rows and columns in the image was obtained, and half of the rows and columns in the image were stored for judging the black edge area. The threshold value was used to judge the pixels in the stored data that are larger than the threshold value, and these were extracted and stored in the black edge area. The upper and lower left and right borders of the black edge area were determined according to the index value and removed. The extra area of the pre-shock image compared to the shake compensation image was cropped. The matching effect diagram after black edge removal and image cropping is shown in Figure 6c. After this step, the camera shake problem was solved and no longer affected the subsequent analysis of the object movement, but the intensity needed to be corrected

### 2.4. Portrait Removal

Before and after an earthquake, people often move in videos taken by monitoring systems, and the movement of these people is misidentified as the movement of objects in the subsequent object movement recognition process, which affects the acquisition of the movement of objects. So, we need to rectify this issue. Traditional image processing algorithms cannot directly identify a human body in an image, so the SAM model [27] and YOLOv8net(YOLOv8) [28] were selected to detect and remove human figures. After the removal of a portrait, a black area will be left on the image, and this area will still be recognized as an object that is moving, so the image needs to be repaired after the portrait is removed.

**Figure 7:**
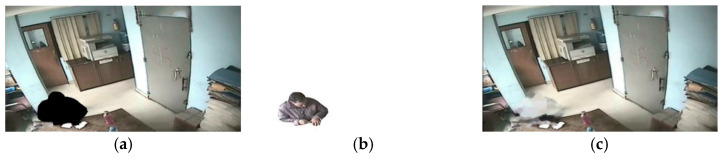
Image restoration after portrait removal. (**a**) Portrait removal image; (**b**) portrait mask image; (**c**) repaired image.

The segment anything model (SAM) is a pre-trained model in the field of image segmentation, released by Meta AI on 6 April 2023. The dataset includes more than 1 billion mask labels and 11 million images. Zero samples can be migrated to new image tasks. The segmentation accuracy of the model even exceeds the partial fully supervised segmentation. However, due to the large amount of pre-training for this model, we chose to run it on a virtual server on Google Colab. YOLOv8net is the latest detection algorithm in the YOLO series and can complete a large number of detections, segmentations, and other tasks with a fast processing speed and a good effect. Only using the SAM to segment the portrait will segment the portrait into multiple masks, such as a head and a coat, and cannot segment the entire human body at one time. Therefore, this study generated the bounding box of the portrait mask according to YOLOv8net and used the SAM to segment the portrait in the bounding box. After portrait recognition and removal by Google Colab, there were black boxes outside the image due to functions of the storage system. The black boxes were detected according to the gray connected-domain algorithm, and image cropping was performed on the image with the portrait removed and the mask image, and grayscale inversion was performed on the cropped image, as shown in Figure 7a,b.

After removing the human figures, a black region was left in place, as shown in Figure 7a. In this study, a texture-guided patch-matching approach combined with multi-scale nested inpainting was employed to restore the removed regions. Firstly, the region to be restored was determined based on Figure 7b. The surrounding information of the restoration region was analyzed, and the average texture features were computed to determine the restoration priority. Then, multi-scale nested inpainting was applied, starting from smaller scales and gradually progressing to larger scales, to restore the regions from which human figures were removed. Finally, an edge refinement algorithm was used to refine the texture at the edges at each scale. The resulting restoration outcome is depicted in Figure 7c.

## 3. Area-Based Object Classification

On the seismic intensity scale for describing the response of objects, indoor objects are classified into several categories: small objects, lightweight furniture, regular furniture, and heavy furniture. However, directly using the area of indoor objects for classification is not accurate due to the depth-of-field effect. To mitigate the influence of the depth-of-field effect, this study divides the images into three regions based on the characteristics of the indoor camera setup and the region clarity: the near region, the central region, and the far region from the camera. Then, objects within these three regions are classified based on their respective areas.

### 3.1. Establishment of Depth-of-Field Region

For capturing more indoor information, surveillance cameras are typically installed on the ceiling, walls, or elevated shelves, with a slight downward tilt. This installation configuration results in objects in the lower part of the camera’s field of view occupying a larger portion of the image, while the upper region appears more distant and presents a smaller size. Consequently, in the captured images, objects in the lower edge region are visually closer to the camera and appear larger in size. Moreover, influenced by the perspective effect and the imaging principles of the camera, objects at the corners of the lower edge appear relatively farther away in the image, while objects at the center of the lower edge appear relatively closer. In this study, an arc-shaped depth-of-field region was established, with the lower edge of the image serving as the boundary. Within a given image, areas closer to the camera exhibit a higher image clarity. The clarity of image regions is determined by considering factors such as the edge density and the average gradient, which serve as criteria for the division of the depth-of-field region.

(5) D(I)=N(I)/(H×W), G(I)=Σ(|Gx|+|Gy|)/(H×W)

In Equation (5), N(I) represents the total number of edge pixels, D(I) denotes the edge density, and H × W represents the image size. |Gx| and |Gy| represent the gradients in the x and y directions, respectively, while G(I) represents the average gradient. The edge density and the average gradient of the three depth-of-field regions were computed. When the edge density is the same, there is a positive correlation between the average gradient and the region clarity. Similarly, when the average gradient is the same, there is a negative correlation between the edge density and the average gradient. The clarity formula was then constructed accordingly.

(6) Q(I)=G(I)/D(I)

In Equation (6), Q(I) represents the clarity measure. The evaluation criterion for determining the percentage of images that meet the clarity requirement follows the order of higher clarity in the nearer region, followed by the central region, and then the farther region. A total of 109 sets of experimental images were selected, and after multiple validations, it was determined that the optimal clarity evaluation is achieved when the line separating the near and central regions is drawn using two points located at 1/10th distance from the lower edge of the image, and the center point of the arc is positioned at 1/4th distance from the lower edge’s central point. Similarly, the line separating the central and far regions is drawn using two points located at 2/5th distance from the lower edge, and the center point is positioned at 3/5th distance from the lower edge’s central point. Ultimately, the established depth-of-field region clarity achieved the best evaluation according to the specified criterion, with 82.8% of the images meeting the clarity requirement.

**Figure 8:**
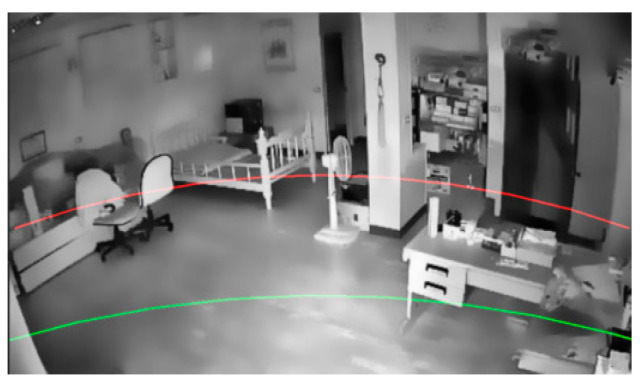
Depth-of-field region segmentation.

The division of the depth-of-field regions is shown in Figure 8. The two points that are 1/10 away from the lower edge of the image are taken as the endpoints, and the point that is 1/4 of the central point of the lower edge of the image is taken as the arc center point. With two points 2/5 away from the lower edge of the image as the end point and the point 3/5 at the center of the lower edge of the image as the center point, the demarcation line of the distance camera is drawn, and the definition of the depth of field area is the best. However, this method of establishing depth-of-field regions may encounter certain issues, such as when a piece of furniture or a heavy object spans multiple regions, and it is necessary to consider the slope of the longest edge of the object. We performed regional compensation for the following scenarios: When the slope approaches one, it indicates that the object is similar to a vertical cabinet, refrigerator, or tall shelf, which tends to block the regions behind it. In this case, the region detected at the lowest point of the object is considered to be the region of the entire tall object, including all objects above it. On the other hand, when the slope is less than 0.9, the target is divided into multiple regions, and the region is redistributed as part of the central target region.

### 3.2. Object Classification

In different indoor scenes and camera angles, the size of objects can vary greatly. This study selected 109 sets (218 images) of pre-earthquake and postearthquake images from various common indoor scenes, such as shopping malls, schools, factories, offices, and residences. Using this annotation method, the areas of 7326 small objects, light furniture, furniture, and heavy items in different depth-of-field regions were calculated. The results were classified according to the depth-of-field regions. However, due to the limited selection of 218 images, the size of each region was extended by 5% to the left and right, respectively. This method produced more robust regional intervals. The classification labels of different types of objects in each region are shown in Table 1.

## 4. Acquisition of Object Movement and Seismic Intensity Assessment

The accuracy of object detection, segmentation, and matching in images is directly influenced by the image processing workflow and the selection of processing methods. This section starts by showcasing the workflow for acquiring object motion information. It then delves into the relationship between object motion patterns and earthquake intensity. Finally, two examples are provided to demonstrate the entire intensity assessment process.

### 4.1. Obtaining Information on Object Movement

**Figure 9:**
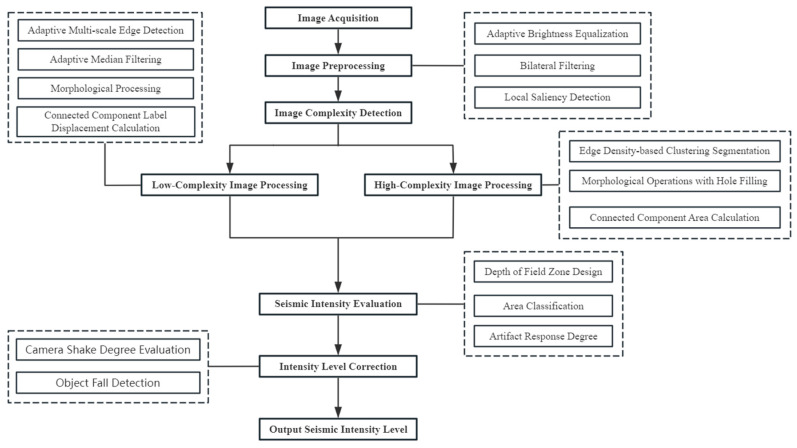
Detection and assessment of intensity through object movement information.

The current study designated areas such as the ground, walls, and ceiling as the background, while considering all other objects as the foreground. By analyzing the movement patterns of the foreground objects, the earthquake intensity was calculated. The detailed steps are visualized in Figure 9.

Upon retrieving the seismic period video from the monitoring system, in order to obtain comprehensive pre- and postearthquake information, the frame preceding the earthquake was extracted as the pre-earthquake image, while the final frame served as the postearthquake image. Brightness compensation was performed using adaptive histogram equalization. Bilateral filtering and local saliency detection were applied to blur the background and enhance the foreground. To select an appropriate image segmentation and matching algorithm, the image power spectral density was employed as a measure of the image complexity. The images are categorized into low-complexity and high-complexity.

(7) PSD=|F−1[H(u,v)·F(I)]|^2/(H×W)

In Equation (7), PSD represents the image power spectral density, H(u, v) is the frequency domain filter function, I is the input signal, and F and F^−1^ represent the two-dimensional fast Fourier transform (FFT) and its inverse transformation (IFFT), respectively. H and W represent the height and width of the image, respectively. In this study, we selected 109 sets of earthquake images from Section 3.2. The power spectral density range of the calculated image indicated a low-complexity image when it was (0, 1540), a high-complexity image when it was (1950, 64,516), and an image with overlapping regions when it was [1540, 1950].

### 4.2. Low-Complexity Image Processing

Images with a power spectral density of less than 1540 are low-complexity images. Low-complexity images usually include scenes such as bedrooms, living rooms, offices, and corridors, and the texture distribution of these images is not very concentrated. The image segmentation algorithm based on edge information can be used to segment the image [29], and the image matching algorithm based on the stable feature region can detect and obtain the state of moving objects. Therefore, the image segmentation based on edge information is designed to judge the movement of objects according to the information obtained from stable feature points.

#### 4.2.1. Adaptive Multi-Scale Canny Edge Detection

In order to obtain the information regarding the movements of the objects in the image, it was necessary to segment the foreground objects in the image more accurately. The adaptive multi-scale canny algorithm has a strong stability and can detect most of the object edges. Through detection processing at different scales, it can also pick up the edges of objects that are difficult to detect and relatively fuzzy. The working principle of the adaptive canny algorithm is firstly to carry out Gaussian filtering on the image and obtain the weighted average of the neighborhood around each pixel to reduce the impact of noise. A sobel operator is used to calculate the gradient amplitude and direction of each pixel in the filtered image. In the gradient direction, non-maximum suppression is performed, only the pixel with the largest value in the gradient direction is retained, and other pixels are suppressed. The double threshold is set, and the edges are divided into three categories: strong edge, weak edge, and non-edge. The strong edge is marked as the true edge, the weak edge is marked as the false edge, and the non-edge pixels are excluded. A scale pyramid is constructed, adaptive canny edge detection is performed on images of different scales, and fuzzy edges are picked up, which are also recorded as weak edges. Finally, the weak edge connected with the strong edge is analyzed for edge connection and connectivity, and the complete edge is formed.

#### 4.2.2. Region-Adaptive Median Filtering

Based on the adaptive multi-scale canny algorithm, there is a large amount of salt-and-pepper noise in the image after edge segmentation. The salt-and-pepper noise is characterized by multiple isolated white noise points in the image, and the salt-and-pepper noise can be eliminated by using the regional adaptive median filtering algorithm. The working principle of the adaptive median filtering algorithm is to first segment the image into nine regions of the same size and calculate the region edge density of the image. The area with denser edges has more and denser salt-and-pepper noise. Therefore, a smaller filter core is used for the area with a higher edge density, and a larger filter core is used for the area with a lower edge density. The image is sorted by pixels in the filter range, the median gray value is detected, and all pixels in the filter are replaced by the median pixel to eliminate all abrupt white noise.

#### 4.2.3. Morphology Operation

After adaptive median filtering, there were still a large number of broken edges and some abnormal holes in the image, which would affect the accuracy of subsequent object detection. In morphological processing, the edge is first expanded to connect the broken edges and some holes are filled, and then the edge is refined by corrosion to prevent excessive expansion from affecting the size of the object. However, the simple use of a single expansion corrosion core will have some problems. Too large a core will cause some small objects to disappear after expansion, and too small a core will cause large edge fractures and cannot be connected. In order to solve this problem, multi-filter kernel nesting processing was used, and the image of all the processing results was obtained according to the size of filter kernel from small to large, step-by-step expansion, and corrosion. The image difference and the image phase sum were, respectively, made for the image after high–low kernel processing, so as to obtain the most suitable processing image and ensure the integrity of main features. The principle of dilatation corrosion is to establish a filter; that is, the result of dilatation is that the pixel expands around the corresponding size, and the result of corrosion is that the pixel shrinks inward of the corresponding size.

After morphological processing, the threshold was set. The stability feature region detection algorithm that was applied in Section 2 detected that the minimum object length was 17 pixels and the longest line width was 13 pixels. Adaptive threshold binarization was used to detect the connection between pixels in the eight neighborhoods and the current pixel. The equivalent labeling algorithm was used to separate different connected domains, extract the area and center point coordinates, and prioritize the object with the most area similarity. If the area was similar, the distance between the feature points was combined to assist judgment. We counted the number of connected domains, i.e., the number of objects identified. For each connected domain, the number of pixels was calculated to approximate the size of the object, and the type of the object was determined according to the size of the object and the depth-of-field area in which it was located. For each connected domain, the coordinates of the center point were calculated to match the objects in the images taken before and after the earthquake. The displacement of the center point between the two objects is the displacement information of the object. Here, the size and number of moving and non-moving objects and the displacement of moving objects were obtained according to the connected domain marker information [30].

### 4.3. High Complexity Image Processing

Images with a power spectral density of greater than 1950 belong to high-complexity images, which are mostly taken from shopping malls, kitchens, and other scenes. The characteristics of such images are that there are a large number of shelves in the images, and a large number of small objects of similar sizes and similar features are placed on the shelves. The edge information is extremely complex, and the feature information is difficult to obtain. The analysis of this kind of image mainly analyzes the movement of objects on the shelves at a low intensity and the fall of objects at a high intensity. Firstly, cluster segmentation was performed on the images taken before and after the earthquake, based on edge information [31], and the images were divided into the background, shelves, and fallen objects. When there was no falling object, the image was differentially detected, depending on whether there was a moving object.

The key principle of edge densified-based clustering segmentation for images is to use the edge density to quantify the pixel distribution information of the edge and classify pixels with similar edge densities into one class by the clustering method, which can effectively gather the edge pixels together and form an effective segmentation region.

The analysis of a high-complexity image mainly detects whether objects fall in the images taken before and after the earthquake, so it is divided into the following two processing processes according to the situation of objects falling. Firstly, the canny algorithm is used to detect the edge of the image, and the number of edge pixels in the neighborhood of the pixel (eight) is calculated for each pixel of the edge image, so that an edge density image can be obtained. Each pixel of the image represents the edge density around it. The image of the edge density is segmented by K-means clustering, and the whole image is divided into three categories according to the edge density. Shelf areas have a high edge density, background areas have a zero edge density, and falling objects have a low edge density. Binary processing was performed on the image after clustering segmentation, and the threshold of the binary processing was set to 57 pixels, the background was set to 255 pixels, and the dropped object and shelf area were set to 0 pixels. When the images taken before and after the earthquake were differentiated, only fallen objects remained in the differential images, but there were a lot of noise and holes in the acquired images of fallen objects. In this case, hole filling and morphological corrosion and expansion should be used to remove the noise and fill the holes, and then the number and area of fallen objects can be obtained by marking the connected domains of the image. The number and size of fallen objects in the high-complexity image were thus obtained. If the fallen object was not detected in the differential image, the cluster segmentation of this set of images was no longer performed, and the images taken before and after the earthquake were directly differentiated after the local significance detection. In this case, only slight movement of objects occured on the shelf, and the brightness transformation was small. Therefore, binary processing at the 25 threshold value was used for the differential image, and the moving object was set as 0 pixels. We set other areas to 255 pixels. The image after binarization was corroded. Finally, the number and size of only the moving objects were obtained by connecting domain labeling and area screening.

After the connected domain was marked, there were some objects with too small an area; these were the noise regions that could not be processed completely. Therefore, it was necessary to perform a low-area screening on the connected domain area before the object movement statistics were calculated to remove most of the noise.

Images with a power spectral density of greater than 1540 and less than 1950 belong to mixed-complexity images, which are mostly taken from the scenes of utility rooms, factories, kitchens, etc. The local areas of such images are complex, while other areas are relatively normal. For such images, the region segmentation based on the power spectral density was performed first, and the images were divided into high-complexity regions and low-complexity regions. According to the corresponding method, the movement of objects in the images of different complexity regions was detected, and the number, size, and displacement of objects in the mixed-complexity images are finally obtained.

At this point, the object movement information in all the complex images was obtained, and the earthquake intensity was evaluated according to the object movement information.

### 4.4. Earthquake Intensity Assessment

The description of object reactions in the seismic intensity table is shown in Table 2.

In Table 2, object reactions occur at intensity levels 3 to 9, while object movement takes place at intensity levels 5 to 9. The objective of this study was to assess the object movement in indoor environments at intensity levels 5 to 9. The quantitative descriptors used in the seismic intensity scale are defined as follows: (a) “few” refers to below 10%; (b) “some” refers to 10% to 45%; (c) “many” refers to 40% to 70%; (d) “most” refers to 60% to 90%; (e) “nearly all” refers to above 80%.

Using the seismic intensity scale descriptors and the defined ranges of quantity terms, an earthquake intensity assessment table based on the percentage of object movement was obtained, with N1, N2, N3, and N4 representing the quantities of moving objects, light furniture, furniture, and heavy furniture, respectively. The total quantities of small objects, light furniture, furniture, and heavy furniture are denoted as N5, N6, N7, and N8, respectively.

By incorporating the object movement information obtained in Section 4.1 into Table 3, the corresponding condition was determined, and the appropriate seismic intensity was assigned. After the initial assessment of the seismic intensity, it was necessary to adjust the seismic intensity estimation based on the object displacement and camera shaking. Minor camera shaking or no shaking during the interval of image capture before and after the seismic event were considered normal conditions. Therefore, the following correction methods were performed in this study:

(1) For seismic intensities of below 7 degrees, check if there is any vertical displacement of objects exceeding three times their length. If such displacement is detected, the objects are considered to have fallen. The seismic intensity should be adjusted to 7 degrees.
(2) If the camera is visibly shaking, increase the seismic intensity by 1 degree.

### 4.5. Example of Earthquake Intensity Assessment

**Figure 10:**
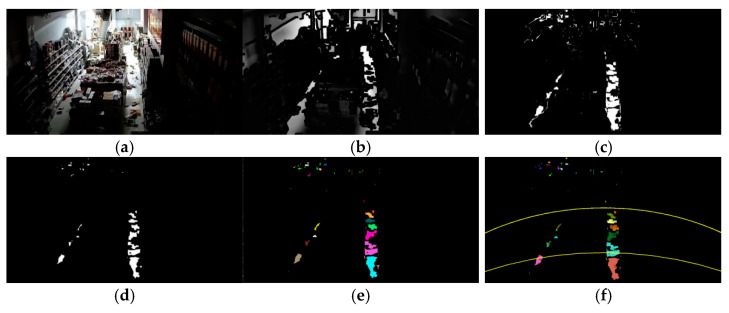
Obtaining object movement information from high-complexity images. (**a**) Original image; (**b**) edge density clustering segmentation; (**c**) differential; (**d**) morphological processing and hole filling; (**e**) connected domain labeling; (**f**) establishment of depth-of-field region.

In this section, two seismic examples are used to demonstrate the overall process of indoor intensity assessment for low-complexity images and high-complexity images. As shown in Figure 10, the power spectral density of the images taken before and after the earthquake is 1430. The process of acquiring object movement information in low-complexity images showed that some small objects moved, light furniture moved, and objects fell in the images. The initial evaluation of the seismic intensity was 7 degree.

The final count of connected components was 12, ranging from 110 to 2317. All the fallen objects were small in size. Therefore, the assessed seismic intensity was 7 degrees, which is consistent with the seismic intensity evaluation results of the team.

**Figure 11:**
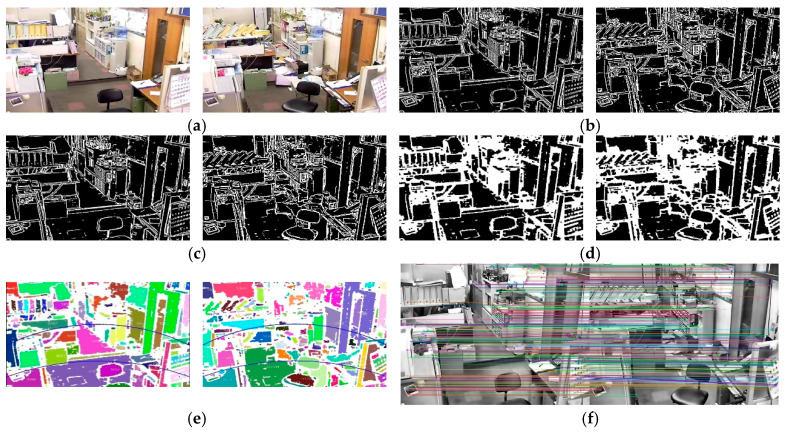
Obtaining object movement information from low-complexity images. (**a**) Low-complexity pre- and postearthquake images; (**b**) adaptive multi-scale threshold segmentation; (**c**) adaptive median filtering; (**d**) morphological processing; (**e**) connected domain labeling; (**f**) stable region feature point matching.

Figure 11 illustrates the process of obtaining object movement information in low-complexity images. Some small objects were observed to move, objects fell, and light furniture moved as well. The seismic intensity was assessed to be 7 degrees.

After removing excessively large background areas and abnormally small outliers, the object movement information obtained is presented in Table 4.

By substituting the object movement information from Table 4 into Table 2, it was determined that the object movement information satisfied the condition k7, corresponding to a seismic intensity of 6 degrees. However, upon further investigation, it was identified that there were three small objects with vertical displacements exceeding three times their length, leading to object falls. As a result, the seismic intensity was adjusted to 7 degrees, which concurs with the manually assessed seismic intensity results.

## 5. Experiment

The content of this chapter is the corresponding simulation experiment and analysis of the seismic intensity evaluation based on object movement information. The main body of the experiment was divided into two parts: The first part of the experiment divided 128 sets of seismic video data collected into four types of scenes: houses, shopping malls, offices, and factories. The data characteristics of each type of scene were analyzed, the seismic intensity was artificially pre-evaluated, the difference in the assessment accuracy between existing methods and the IESI method was compared, the reasons for the wrong assessment of different methods were analyzed, and the advantages of the IESI method intensity assessment were verified. In the second part of the experiment, the video data of 11 groups of the Dezhou earthquake of magnitude 5.5 in Shandong Province were collected from video websites, and the evaluation intensity of the IESI method was compared with the real seismic intensity to verify the applicability of the IESI method in low-intensity areas. The chapter concludes with a brief introduction to the possible applications of the IESI method.

### 5.1. Experimental Preparation

**Figure 12:**
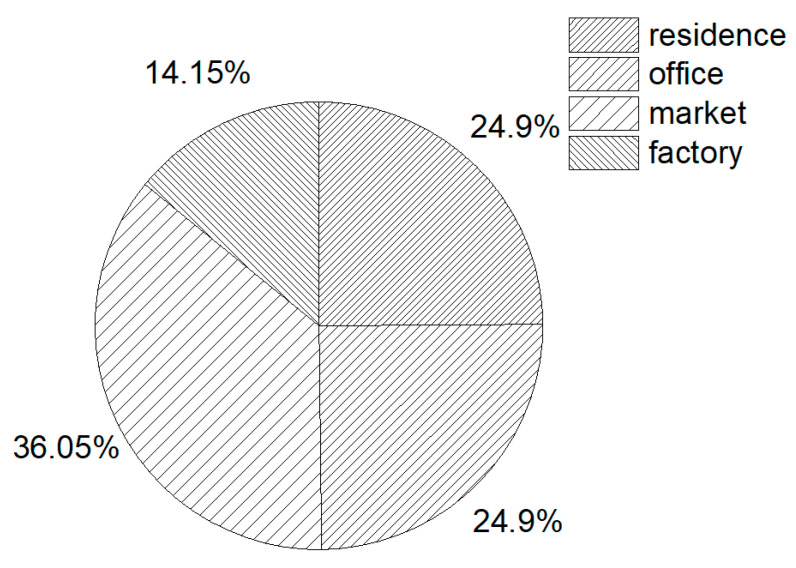
The overall distribution chart of the data.

The video data used in the experiment are real earthquake videos obtained from major video websites at home and abroad (Baidu, Bilibili, YouTube, etc.). The intensity divisions covered by the data include the following: the Dezhou earthquake of magnitude 5.5 in Shandong Province with a low intensity; in the medium-intensity area, the Luding earthquake of Sichuan, magnitude 6.8, and the Mexico earthquake, magnitude 7.0; in the high-intensity area, the earthquake in Turkey–Syria, magnitude 7.8, and the earthquake in Tokyo, Japan, magnitude 9.0; and other earthquakes of various intensities. Indoor scenes included common scenes such as residences, shopping malls, factories, and offices. The video content included unusual lighting, complex scenes, moving objects that are rare and difficult to detect, camera shaking, people moving, and other common situations. The data selection included the whole intensity interval, common scenes, and common situations, which proves the universality of the method. The specific data distribution is shown in Figure 12.

Due to the slight differences in the instrument response descriptions and earthquake intensity evaluation standards among different countries and regions (namely the instrument response description of the Japan Meteorological Agency Earthquake intensity scale, the European Earthquake intensity scale, and the McCully Earthquake intensity scale), we first mapped these to the instrument response description of the China earthquake intensity scale, and then the intensity was finally determined according to the corresponding results with the China earthquake intensity scale. In order to ensure the objectivity of the intensity pre-assessment results, the seismic intensity of the artifact response of each image was evaluated by all the team members. In order to facilitate their display and analysis, for each group of images taken before and after the earthquake, we marked the movement percentage of various objects on the left side, the seismic intensity evaluated by the team on the upper right side, and the seismic intensity evaluated by this method on the lower right side.

The IESI method was compared with the IDEAS and PSAB methods. The IDEAS approach is used to estimate the seismic intensity experienced indoors based on the area variance between moving objects and non-moving objects before and after an earthquake. In order to evaluate the intensity, the PSAB method needs to be adjusted to some extent. The PSAB method detects the movement of objects by laying sensor lines. When the movement percentage of objects is greater than 50%, the PSAB method determines the damage. The accuracy of the PSAB depends on whether it can accurately distinguish seismic intensity VI (the dividing line between whether a large earthquake damage occurs in the seismic intensity scale). In other words, when the PSAB method estimates that there is damage, and the earthquake intensity is greater than or equal to VII degrees, the assessment is correct.

In order to improve the visualization effect of the data, 3 groups of images were selected for each scene, and a total of 12 groups of images were displayed. On the left side of the image data, the movement percentage of different types of objects was added in the order of small objects, light furniture, furniture, and heavy furniture; on the right side, the seismic intensity evaluated by other methods was added in the order of the initial assessment, the IESI method, the IDEAS method, and the PSAB method.

### 5.2. Comparative Experiment

In order to verify the applicability of the IESI method in different scenarios, the IESI method, PSAB method, and IDEAS method were used to evaluate the seismic intensity of residential scenes, shopping mall scenes, office scenes, and factory scenes and analyze the causes of wrong assessments.

#### 5.2.1. Residential Scene

The scene selected for this part of the experiment was the residential environment. In the residential scene, there are not many lights on at night, and the power system is relatively fragile, so the abnormal brightness is the most obvious, and brightness compensation and image enhancement need to be performed several times. Only the detection results show that a large number of objects are often placed in the kitchen and storage room scenes, so they are high-complexity images, and other scenes are mostly low-complexity scenes. The table showing the analysis is shown in Table 5, and the accuracy rate of the intensity evaluation by different methods is shown in Table 6.

In Table 5, the left side of the table shows the percentage of the movement of different types of objects; from top to bottom are the percentage of the movement of small objects, light furniture, and heavy furniture. On the right side of the table, from top to bottom, the pre-evaluation intensity, the IESI evaluation intensity, the IDEAS evaluation intensity, and the PSAB evaluation of earthquake damage are shown, respectively.

As can be seen from Table 6, when the intensity is lower than 6 degrees, the accuracy of all the methods is 100%, while when the intensity is higher than VII degrees, the assessment results of the PSAB method are all wrong, so the assessment of indoor earthquake damage by the PSAB method is incorrect. As long as heavy furniture is moved, even if the percentage of the object movement is not high, there is still a lot of earthquake damage. This result further confirms that it is not sufficient to rely solely on the percentage of object movement to assess earthquake intensity. The IDEAS method is also less accurate when evaluating an intensity of above VII. This result further confirms that the correction ability of area variance is insufficient at a high intensity. Therefore, it was necessary to make some intensity corrections after the intensity evaluation.

To sum up, the accuracy of the IESI method in assessing seismic intensity was much higher than that of the PSAB method and the IDEAS method in the tested residential scenarios. The IESI method’s confidence is derived from the pre-processing of anomalies, using the percentage of the movement of different object types to estimate the seismic intensity, and the IESI method also makes some intensity corrections based on the falling of objects and the shaking of cameras.

#### 5.2.2. Shopping Mall Scenario

The scene selected in this part of the experiment was the shopping mall environment. The object distribution feature of the shopping mall scene was that a large number of shelves are placed, and there are a large number of objects on the shelves, so the images in the shopping mall scene are high-complexity images. The images displayed for analysis are shown in Table 7, and the intensity assessment accuracy of different methods is shown in Table 8.

As can be seen from Table 7, there are only objects falling in 3 groups of images, and heavy furniture moving in another group of images. As can be seen from Table 8, in the shopping mall scene, the accuracy of the PSAB method is still very low when processing images above the VII degree. At the same time, at the intensity of 7 degrees, the performance of the IDEAS method is poor. A large number of moving objects were detected during processing, and the intensity was assessed to be 9 degrees, which was inconsistent with the actual situation.

In the tested shopping mall scenario, the IESI method was much more accurate in assessing seismic intensity than the PSAB method or the IDEAS method. Because the shopping mall image is a complex image, the accuracy of acquiring the movement of the object is greatly improved by the cluster-based processing of this kind of image. According to our experimental results, the IESI method achieved 100% accuracy in all the mall environments tested.

In Table 7, The left side of the table shows the number of movements of small objects, light furniture, and heavy furniture from top to bottom. On the right side of the table, from top to bottom, the pre-evaluation intensity, the IESI evaluation intensity, the IDEAS evaluation intensity, and the PSAB evaluation of the earthquake damage are shown, respectively.

#### 5.2.3. Office Scenario

The scene selected in this part of the experiment is the office environment, which is placed with tables, chairs, and folders and is easily affected by occlusion. Therefore, an appropriate compensation area is required for the depth-of-field estimation and area classification to reduce the influence of object occlusion. The images displayed for analysis are shown in Table 9, and the intensity assessment accuracy of different methods is shown in Table 10.

As can be seen from Table 9, there was an error in the IESI method’s evaluation of intensity VI, and the movement of light furniture appeared in the image. However, due to the occlusion of objects, the IESI method mistakenly identified the movement of light furniture as the movement of small objects when detecting the movement of objects. The seismic intensity was incorrectly assessed as V, but the actual seismic intensity was VI. There was also an error in the evaluation of intensity IX, in which large objects moved in the image. However, due to the large number of objects in the refrigerator, the refrigerator was identified as several small objects in the object recognition process, and the movement of large objects was identified as the fall of small objects. The earthquake intensity was incorrectly assessed as VII, but the actual earthquake intensity was IX. When object occlusion occurs and a large number of small objects are placed on top of large objects, the image processing is not good enough.

#### 5.2.4. Factory Scenario

The scene selected for this part of the experiment was the factory environment. The images displayed and analyzed are shown in Table 11, and the accuracy of the intensity evaluation by different methods is shown in Table 12. Some areas of the factory scene are empty, and some areas have a lot of debris placed there, so most of them are mixed-complexity images and low-complexity images.

It can be seen from Table 11 that the IESI method made an error in evaluating the intensity of 7. When detecting the fall of the crossbar, the IESI method did not recognize the fall of the crossbar as the fall of an object because the length of the crossbar was too long, resulting in an error in evaluation.

At the same time, taking the 5.5-magnitude earthquake that occurred in Dezhou City, Pingyuan County, Shandong Province, at 2:33 am Beijing time on 26 August 2023 as an example, the IESI method evaluated the earthquake intensity range of Dezhou City, Shandong Province as V, VI, and VII, which was consistent with the intensity range published by the network. The results show that the IESI method can provide assistance for seismic intensity assessment in low-intensity areas.

In summary, the PSAB and IDEAS methods perform well when the percentage of moving objects is very low or very high. The reason for this is that it is easy to perform a disaster assessment, and the area variance correction for the percentage of moving objects will not have a large impact if the percentage of moving objects is very low, and it is highly likely that there is no damage inside the building. However, the accuracy of the PSAB and IDEAS methods decreased significantly when the intensity was higher than VII. The IESI method does not have this problem because it evaluates the seismic intensity based on the percentage of movement of different types of objects while selecting different segmentation matching algorithms. However, the IESI method has some problems dealing with occlusion and extremely long objects.

## 6. Conclusions

This paper focused on the use of appropriate image processing algorithms and procedures to accurately obtain object movement information. Taking earthquake video data from Dezhou, Shandong, Luding, Sichuan, and the Turkey–Syria earthquake as samples, we used image processing algorithms to extract motion information from pre-earthquake and postearthquake images, including the number, size, and displacement of objects. Based on this data, we determined the target response level and assessed the earthquake intensity using the corresponding earthquake intensity table and corrected the assessed intensity. The assessment range was V-IX degrees, which is mainly used for intensity assessment in low-intensity areas of perceptible earthquakes and destructive earthquakes, providing technical support for postearthquake rescues, and intensity map drawing. The main conclusions of the study are as follows:

1. Preprocessing techniques were used to process abnormal images; low-quality images were rejected, and camera shake was compensated for based on stable feature information, and area calculations were performed based on depth effects to construct depth evaluation regions. Different motion target detection methods were designed according to the complexity of the image. The percentage of the movement of different targets was used instead of the area variance to assess intensity levels.
2. During the intensity assessment and correction, to ensure the reliability of the assessment, an indoor intensity assessment table was abstracted based on the description of object responses and quantifiers in the intensity table. The intensity was assessed based on the movement of different types of objects. After the intensity assessment, the earthquake intensity estimation was corrected based on the situation of object dropouts and camera shake.
3. Compared to existing methods, such as the PSAB and IDEAS methods, this method combines edge density clustering segmentation with image morphological processing to handle high-complexity images. The experimental results show that the proposed algorithm is superior to the existing algorithms, especially in cases of camera shake and significant object movement.

## Figures and Tables

**Table 1:** Area-based object classification in various regions.

Region	Small Objects	Light Furniture	Furniture	Heavy Furniture
Distant areas	[141, 1131)	[1131, 3741)	[3741, 9281)	[9281, 37,291]
Centered area	[434, 2443)	[2443, 6646)	[6646, 16,023)	[16,023, 95,396]
Near region	[236, 3529)	[3529, 9386)	[9386, 28,823)	[28,823, 129,167]

**Table 2:** Description of instrument response.

Seismic Intensity	Instrumental Reaction
III	Slight movement of suspended objects.
IV	The hanging object swings noticeably and the utensils make a noise.
V	The suspended object shakes significantly, with a small number of small items on the shelf, some heavy or unstable objects placed on the top shaking or overturning, and water shaking and overflowing from the filled container.
VI	A small amount of light furniture and objects move, and a small number of heavy objects on the top overturn.
VII	Objects fall off the shelf, most heavy objects on the top overturn, and a few pieces of furniture overturn.
VIII	Except for heavy furniture, most indoor items are dumped or displaced.
IX	Most indoor items are dumped or displaced.

**Table 3:** Quantifying the degree of instrument response to assess seismic intensity.

Condition	Movement of Different Types of Objects	Seismic Intensity
k1	N4 greater than 0	9
k2	N3/N7 ∈ [0.6, 1]	8
k3	N3/N7 ∈ [0.45, 0.6) and (N2 + N3)/(N6 + N7) ∈ [0.6, 1]	8
k4	N3/N7 ∈ (0, 0.45)	7
k5	N2/N6 ∈ [0.6, 1]	7
k6	N2/N6 ∈ [0.45, 0.6) and N1/N5 ∈ [0.6, 1]	7
k7	N2/N6 ∈ [0.1, 0.45)	6
k8	N2/N6 ∈ [0, 0.1) and N1/N5 ∈ [0, 0.45), When the two are different, they are 0	5
k9	N1/N5 ∈ [0.45, 1]	6

**Table 4:** Object movement information in low-complexity images.

Mobile Information	Small Objects	Light Furniture	Furniture	Heavy Furniture
Mobile quantity	39	4	0	0
total	66	12	7	2
Movement percentage	0.591	0.333	0	0

**Table 5:** Evaluation showcase of selected residential scene images.

Movement Status	Pre-Earthquake	Postearthquake	Intensity Evaluation
61.8%	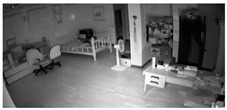	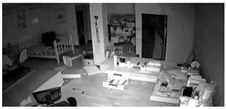	IX
50%			IX
33.3%			VII
50%			Damaged
10.5%	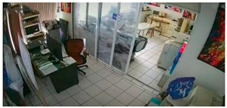	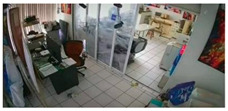	V
0%			V
0%			V
0%			Undamaged
8.2%	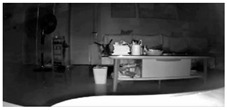	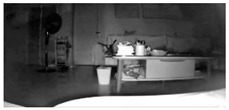	V
0%			V
0%			V
0%			Undamaged

**Table 6:** Accuracy of evaluation methods in residential scene.

Method	V	VI	VII	VIII	IX
PSAB	100%	100%	0%	0%	33.3%
IDEAS	100%	100%	50%	0%	33.3%
IESI	100%	100%	100%	100%	100%

**Table 7:** Evaluation showcase of selected shopping mall scene images.

Movement Status	Pre-Earthquake	Postearthquake	Intensity Evaluation
Thirteen	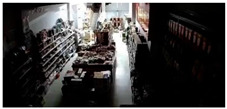	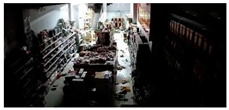	VII
——			VII
——			VI
——			Undamaged
Six	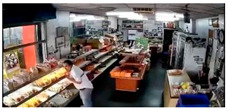	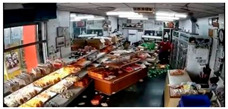	IX
one			IX
——			VII
one			Undamaged
six	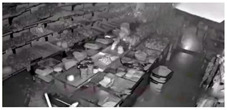	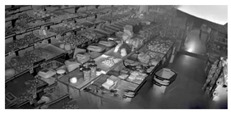	VII
——			VII
——			V
——			Undamaged

**Table 8:** Accuracy of evaluation methods in shopping mall scene.

Method	V	VI	VII	VIII	IX
PSAB	100%	none	14%	none	0%
IDEAS	100%	none	29%	none	100%
IESI	100%	none	100%	none	100%

**Table 9:** Evaluation showcase of selected office scene images.

Movement Status	Pre-Earthquake	Postearthquake	Intensity Evaluation
57.1%	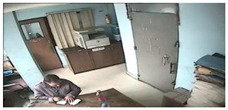	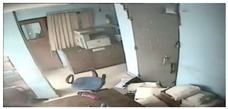	VII
60%			VII
0%			VII
0%			Undamaged
41.1%	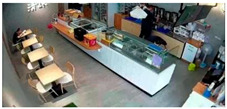	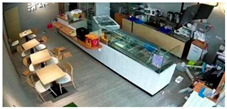	IX
10%			VII
33.3%			VI
0%			Undamaged
20%	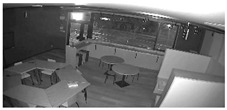	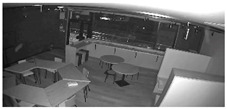	VI
0%			V
0%			V
0%			Undamaged

**Table 10:** Accuracy of evaluation methods in office scene.

Method	V	VI	VII	VIII	IX
PSAB	100%	100%	16%	0%	0%
IDEAS	100%	33%	68%	66%	33%
IESI	100%	66%	100%	100%	50%

**Table 11:** Evaluation showcase of selected factory scene images.

Movement Status	Pre-Earthquake	Postearthquake	Intensity Evaluation
22.7%	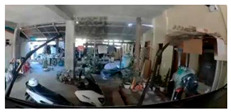	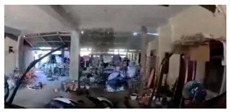	IX
12.5%			IX
0%			VII
33.3%			Undamaged
0%	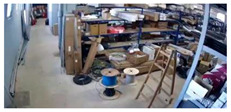	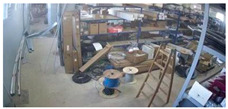	VI
20%			VII
0%			VI
0%			Undamaged
28.6%	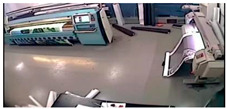	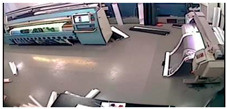	V
0%			V
0%			V
0%			Undamaged

**Table 12:** The accuracy of evaluation methods in a factory scene.

Method	V	VI	VII	VIII	IX
PSAB	100%	100%	0%	0%	33.3%
IDEAS	100%	75%	50%	40%	0%
the IESI method	100%	75%	100%	100%	100%

## Data Availability

The seismic intensity assessment dataset generated during this study is available in the Zenodo repository at https://doi.org/10.5281/zenodo.15233920 (v1.2), under a CC BY 4.0 license.
